# The Impact of Knowledge Management Process and Intellectual Capital on Entrepreneurial Orientation and Innovation

**DOI:** 10.3389/fpsyg.2022.772668

**Published:** 2022-05-20

**Authors:** Qi Yu, Sumaira Aslam, Majid Murad, Wang Jiatong, Nausheen Syed

**Affiliations:** ^1^School of Foreign Languages, Hebei University of Technology, Tianjin, China; ^2^Faisalabad Business School, National Textile University, Faisalabad, Pakistan; ^3^Department of Management Sciences, Muhammad Nawaz Sharif University of Engineering and Technology, Multan, Pakistan; ^4^College of Teacher Education, Zhejiang Normal University, Jinhua, China; ^5^Department of Business Administration, Government College Women University, Faisalabad, Pakistan

**Keywords:** knowledge management process, intellectual capital, entrepreneurial orientation, innovation, IT industry, Pakistan

## Abstract

Innovation is considered to be a dominant cause for sustainable business success. Knowledge management and intellectual capital are powerful tools to promote innovation in the organization. Therefore, this study aims to explore the influence of knowledge management process and intellectual capital on innovation with the mediating effect of entrepreneurial orientation and moderating role of leader education level. Data were collected from a sample of 393 IT firms listed in the Pakistan Software Houses Association and applied a partial least squares structural educational modeling (SEM) technique. The results show that the knowledge management process and intellectual capital have a positive effect on innovation. Moreover, the study confirms that entrepreneurial orientation partially mediates the relationship between knowledge management and intellectual capital on innovation. Furthermore, the moderation effect of a leader’s education was confirmed for the knowledge management-innovation relationship whereas, the moderation effect of the leader’s education on intellectual capital and innovation relationship was insignificant. Recommendations for practitioners and future research directions were also discussed.

## Introduction

Globalization and growing technological changes have forced organizations to adopt more creative and innovative work patterns (Al-Jinini et al., 2019; Ruiz-Ortega et al., 2020). Individuals and organizations bring new ideas to markets frequently because they are facing competition at both domestic and international levels. Thus, for survival in such globally competitive markets, innovation has become a major challenge and is considered to be the key element for business success (Buenechea-Elberdin et al., 2017). It is pertinent to mention that innovation displays a crucial part in the development of a company and helps companies to gain a competitive advantage over their rivals (Hassan et al., 2018; Alvino et al., 2020). Flanigan et al. (2017) define knowledge management as the process of creating, increasing, sharing, and transforming the knowledge and information of an organization. Li et al. (2019) view innovation as a vital factor for long-term business success. The innovation of a firm depends upon the current competencies and capabilities to enhance or convert the older technology into new technology (Rasheed et al., 2017). A successful and sustainable innovation process is dependent on intellectual capital and knowledge management as resource-based theory (RBT) states that organizational innovation depends upon the internal capability of a firm, such as knowledge, ability, and employees’ skills, and proper management of the knowledge and intellectual capital (Cabrilo and Dahms, 2018). In the current intense competitive world, the establishment of knowledge-based resources in organizations is vital for successful organizational operations, and to knowing what information is more critical to the organizational success than what it owes (Asemokha et al., 2019).

Intellectual capital is considered the best powerful competitive weapon and a very precious asset of an individual (Iqbal et al., 2018). Intellectual capital is a major source for a company for achieving innovation in processes, products, and getting an edge of competitive advantage in the marketplace (Li and Yu, 2018). An innovation in an organization is mainly dependent on different facets of intellectual capital (Dabić et al., 2018) comprising human, organizational, and social capital. The real value of a company lies in finding the hidden brainpower (intellectual capital) which provides a competitive advantage to improve the market value and financial performance (Zhai et al., 2018). Furthermore, Naqshbandi and Jasimuddin (2018) emphasize that it is important that critical knowledge possessed by the organization should be stored in an appropriate forum for long-term innovation; furthermore, to attain a sustainable comparative advantage, the organization should also ensure creativity. Such types of knowledge strategies and policies are necessary to attain better quality knowledge management to enhance sustainable comparative innovations since there is little research connecting the intellectual capital and knowledge management with innovation, particularly in the context of a collectivistic country as these conceptions are earlier developed and tested in Latin America and Western culture (Migdadi et al., 2017; Le and Lei, 2019).

Entrepreneurial orientation is a vital source for the success of an organization. Entrepreneurial orientation is extracted into entrepreneurship; it takes all action, adopted techniques, and decision-making activities that encourage starting a new business (Tajeddini et al., 2020). Moreover, entrepreneurial orientation also boosts entrepreneurial activities. Literature evidence that there is a positive association between intellectual capital and entrepreneurial orientation (Zhai et al., 2018; Al-Jinini et al., 2019). Entrepreneurship serves as the backbone of a country since it has an essential role in improving the economy of the country (Li et al., 2020). It necessitates studying those resources, which cannot be quickly and easily imitated by revivals, such as the intellectual capital of the organization and entrepreneurial orientation. Moreover, as discussed above, an effective knowledge management process based on its inimitability leads toward sustainable competitive advantage in the marketplace and aids differentiation up to a sustainable level and enhances innovation. Proper knowledge management enhances organizational capabilities to utilize resources of firms properly to exploit new opportunities arising in the market. Therefore, knowledge has become a significant foundation of entrepreneurial orientation that energizes the strategic orientation of a firm and enable it to adapt to environmental changes and react to trendy opportunities (Li et al., 2019).

According to McDowell et al. (2018), innovation is explained as the organizational capability to launch novel goods and services and these novel goods and services are considered as innovation because they present the capability of an organization to adjust according to market changes. In a similar pattern, Masa’deh et al. (2018) explain that entrepreneurial orientation leads to superior performance because it makes owners and managers more vigilant toward the adaptation of the latest market demands and changes, thus promoting innovation. However, few types of research within the existing body of knowledge management process and intellectual capital testified to the positive association between entrepreneurial orientation and innovation in the context of developed countries (Al-Jinini et al., 2019).

The literature-based evidence has been spotted where the relationship between grassroots innovation and entrepreneurial success was fully mediated by entrepreneurial orientation (Singh et al., 2019). So, in a Pakistani business perspective, the current study has the objective of unfolding the intervening role of entrepreneurial orientation in the relationship between intellectual capital, knowledge management, and innovation. Literature has well defined the integral role of leadership education across the organizations. It has been established that leadership success is the cause of higher education; therefore, antiquities discussed that the innovation of a firm depended upon the education level of leaders (Silva, 2014). Similarly, higher education levels of leaders may change the intensity of the relationship between intellectual capital and knowledge management with innovation as employees’ abilities and organizational ability to process knowledge, leading to increased innovation (Cabrilo and Dahms, 2018). Therefore, to fill this gap, this study investigates the moderating effect of leaders’ education on the relationship between knowledge management, intellectual capital, and innovation.

This study contributes to the literature on knowledge management, intellectual capital, and innovation because this is the first study that links knowledge management and intellectual capital with innovation through the mediating effect of entrepreneurial orientation and the moderating role of a leader’s education in the IT (hi-Tec) sector of Pakistan. After all, prior studies explore different outcomes of intellectual capital and innovation (Han and Li, 2015; Obeidat et al., 2017), while little is known about the relationship between knowledge management and innovation (McDowell et al., 2018; Alrowwad et al., 2020). Therefore, this study is the first that explores the level of innovation in developing countries like Pakistan because Pakistan stands at a very low pace in entrepreneurship and innovation compared to its neighbor countries in Asia (Poblete and Mandakovic, 2020; Tok, 2020). It is pertinent to mention that a prior study has been conducted in SMEs, manufacturing, and pharmaceutical sectors (McDowell et al., 2018), but the current study aims to testify to the relationship in the IT (hi-Tec) sector because the IT industry plays a crucial role in enhancing the innovation in the era of digitalization, and many prior studies call for enhancing the understating of the innovation.

Based on the above-mentioned research inputs, this study aims to identify these research gaps in the conceptualization of knowledge management process on entrepreneurial orientation and innovation with the moderating role of leaders’ education. Therefore, within the developed research model, the study investigates the following research objectives:

RO1: To identify the impact of knowledge management process and intellectual capital on entrepreneurial orientation and innovation.

RO2: To assess the mediating effect of entrepreneurial orientation in the relationship between knowledge management process and intellectual capital on innovation.

RO3: To examine the moderating role of leader education in the relationship between knowledge management process and intellectual capital on entrepreneurial orientation.

The remaining part of the article is ordered as follows: Section “Theoretical Framework” discusses the theory and hypotheses of development. The methodology and data are presented in section “Materials and Methods,” while empirical results are reported in section “Results,” and discussion and conclusion are explained in section “Discussion and Conclusion.” Theoretical and practical implications are explained in section “Implications.”

## Theoretical Framework

### Theoretical Support

The foundation of the current study was established on entrepreneurial orientation and the resource-based view (RBV). This explains how entrepreneurial efforts and organizational internal resources should be focused on because these are the center of attention in the RBV theory and concepts of entrepreneurial orientation (Bacq and Eddleston, 2016; Ziyae and Sadeghi, 2020). The RBV conceptualizes the firm as a bundle of resources and highlights the importance of resources to generate an added value to the firm (Ziyae and Sadeghi, 2020). To sustain performance over time, firms must possess or develop unique resources, i.e., resources that cannot be easily copied by other firms (intellectual capital) and proper knowledge management. The extended perspective of the RBV includes all types of resources accessed by a firm (Burvill et al., 2018), while the appropriate realization of entrepreneurial orientation is beneficial not only for the effective mobilization of resources but also coordinates the firm to interconnect stakeholders within or among the firms like governmental agencies and business partners (Nagano, 2019). The different entrepreneurial orientation dimension focuses on the attitude of enterprises toward risk, which is the key to decision-making, research and development, and product/service launch (Korobov et al., 2017). The RBV recommends forming a resource bundle that is unique enough to create the foundation for getting a competitive advantage for a firm that is difficult to be imitated by the competitors of the firm (Pee and Kankanhalli, 2016).

Prior studies argue that companies that are increasingly developing their knowledge management processes and intellectual capital tend to improve their entrepreneurial orientation over time and eventually promote innovation in firms that are supposed to explore in this research (Al-Jinini et al., 2019; Alvino et al., 2020). Based on the discussion on RBV, the specific intentions of this research are to explore the following factors: (1) knowledge management processes to accumulate and utilize the knowledge in a time to support entrepreneurial orientation; (2) a firm’s intellectual capital can appropriately cope up with different uncertainties inherited in such highly variant activities as RBV believes that the intellectual capital provides the basis for further development and enhancement of employee’s capacities through continuously emphasizing on collaborative learning opportunities; (3) entrepreneurial orientation enhances the innovation capacity of a firm because it helps to identify the prevailing opportunities, firm’s capacity to exploit these opportunities, and its capability to adapt these opportunities appropriately. Furthermore, Kianto et al. (2017) argue that innovation has been recognized as an important driver of economic growth and often enables organizations to provide higher quality products and services at lower prices; and (4) the role of a leader’s education level in exercising entrepreneurial orientation to exploit innovation infirm.

### Hypothesis Development

#### Linking the Knowledge Management and Innovation

Regional economic growth and innovation knowledge are considered as basic inputs. In local and international markets, knowledge is an integral resource to improve competitiveness (Teixeira et al., 2019). According to Payal et al. (2019), knowledge has two core types, those that are explicit and tacit. Antunes and Pinheiro (2020) suggest that it is easy to store explicit knowledge, and it can be conceptualized on the other side; it is very difficult to communicate, formalize, and share the tacit knowledge because it belongs to the personal trait. To organize, acquire, capture, and communicate both explicit and tacit knowledge of employees through an organizational systematic framework requires knowledge management. To maximize the organization’s knowledge, other employees would take advantage of this knowledge in the most effective way (Ode and Ayavoo, 2020). Silvianita et al. (2020) developed knowledge management features as a dynamic idea that blended a system approach (Bashir and Farooq, 2019).

Every sort of knowledge that is acquired, developed, and disseminated must be accompanied by authorization and knowledge storage; otherwise, a company runs the risk of unintentionally forgetting to acquire knowledge (Hussinki et al., 2017). The tacit knowledge is the type on which the organizational innovation process highly depends. By changing broad knowledge into specific knowledge, new and useful knowledge is developed and translated into goods, services, and processes (Shiranifar et al., 2019). Attia and Essam Eldin (2018) discussed that it is important to stress that an organization will put a lot of effort into remaining inventive, assuring creativity, and achieving long-term competitive advantages if its vital knowledge is not stored properly. A specific system or website is also essential to achieve enhanced knowledge management results for many types of inventions (Singh et al., 2019).

Knowledge policies and strategies, according to the literature on knowledge management, have an impact on a company’s ability to innovate and persist (Naim and Lenka, 2017). The antiquities shed light on obtaining market intelligence, which is critical for fostering novelties that best meet customer wants. López-Nicolás and Meroño-Cerdán (2011) studied Spanish enterprises and found that a knowledge management approach improves the inventive capacities and competencies of a firm, resulting in long-term performance. On the foundation of proceedings, the following hypothesis has been framed:

**H1:** There is a positive relationship between knowledge management and innovation.

#### Linking the Intellectual Capital and Innovation

Intellectual capital is a collection of solid reserves including institutional explicit knowledge, skills, experience, technology, and contacts that enable an organization to compete in the sector (Liu, 2017a). Roos (2017) defines the concept of intellectual capital as a “multifaceted and heterogeneous concept.” Intellectual capital is “knowledge that is of value to an organization (Kianto et al., 2017). Human capital, relational capital, and structural capital are the three categories of interrelated intellectual capital components that scholars study (Li and Liu, 2018). The ability of an organization to create value and compete is mainly determined by its intellectual capital and ability to innovate. This research looks at the impact of several sorts of intellectual capital, like social and human, social capital, on the innovative outcome. Every human being can be a useful asset or a burden to an organization (Xu and Wang, 2018). Human capital is a combined resource derived from workforce tacit knowledge, capacities, and skills (Ginesti et al., 2018). The scientific results of many studies offer compelling reasons for the importance of human capital in the process of innovation since the value and originality of human knowledge are critical to the process (Yong et al., 2019). Furthermore, much more scientific evidence has emerged in the last 15 years demonstrating that the potential of an organization to innovate is closely related to its human capital and that there is a significant relationship between human capital and innovation (Amin and Aslam, 2017; Obeidat et al., 2017; Smriti and Das, 2018).

The connection and collaboration among individuals who share their ideas result in social capital (Al-Jinini et al., 2019). Buenechea-Elberdin et al. (2017) elaborated that the importance of social capital in encouraging adoption and overcoming the limits of a lack of financial, human, and natural capital cannot be overstated. Dabić et al. (2018) stress the importance of social capital in the creation of innovation. Moran (2005) analyzes the effect of both relational and conceptual involvement of social capital on performance management, emphasizing the importance of relational involvement for enhancing innovation capability, taking into account the extent of personal commonality (proximity), and the notion of trust in the relationship. Liu (2017b) contended that innovation is essentially a collective effort in which social capital plays a critical role. Additionally, previous research suggests that intra-organizational knowledge sharing (social capital) promotes company innovative behavior since it fosters innovation and inspires knowledge and innovation (Mehralian et al., 2018; Barrena-Martinez et al., 2019). Tiwari and Vidyarthi (2018) described why the launch strategy for new products is positively correlated with social capital.

In summary, human capital and social capital play an active role in the innovation activities of a firm. As intellectual capital has primarily focused on the utilization of resources in the organization (Kamukama and Sulait, 2017), the RBV emphasized the creation and utilization of organizational resources and the perspective of intellectual capital effectively and efficiently, taking the maximum benefits and values from the existing resources and capability (Korobov et al., 2017). Moreover, human resource management (HRM) scholars’ resource-based theory serves as a strategy for an organization that has human capital, which is the essential element of innovation (Yong et al., 2019). The perspective of resource-based theory covers tangible and intangible resources like human capital and social capital as the major force of innovation (Burvill et al., 2018). Based on this discussion, the following hypothesis is established;

**H2:** There is a positive relationship between intellectual capital and innovation.

#### Linking Knowledge Management and Entrepreneurial Orientation

A firm’s knowledge depends mainly on its internal and external environment and the environmental dynamism is a source of opportunity and resources (Bojica and Fuentes, 2012). The company’s equity knowledge is a great resource for entrepreneurial ventures, assisting firms in determining the value of recently discovered opportunities and advising on how to better represent the new markets. It also creates awareness of market imbalance that entrepreneurial activities could decrease (López-Nicolás and Meroño-Cerdán, 2011; Rosenbusch et al., 2011). Consumers’ wants are often difficult to articulate; thus the business must share some of the same tacit knowledge as its customers in order to understand their needs and develop new products (Kamukama and Sulait, 2017). As a result, knowledge management is crucial not only for identifying opportunities but also for maximizing them.

As a result, a company that does not gain information from its peer relationships may miss out on the possibilities to better exploit entrepreneurial prospects, reducing the impact of these opportunities on performance (Bojica and Fuentes, 2012). According to the literature, having a lot of knowledge-based resources and a lot of network capacity are vital for turning entrepreneurship into excellent performance (Moustaghfir et al., 2013). On the one hand, since market and technology knowledge strengthen the relationship between entrepreneurial orientation and firm performance, a collection of knowledge-based capabilities can be used to identify and exploit entrepreneurial possibilities. Furthermore, businesses with better prior knowledge find and exploit more connected opportunities. As a result, we can conclude that having a high degree of market and technology expertise is a critical condition for attaining the goals of corporate entrepreneurship. Furthermore, some scholars argue that having a strong knowledge base, as well as increasing it through organizational learning, may be a key determinant of the relationship between entrepreneurial activities, business innovation, and performance (Monteiro et al., 2019; Ruiz-Ortega et al., 2020). Such activities can play an essential role in the firm’s ability to be entrepreneurial orientation and improve performance. So, based on the existing literature, this study hypothesized that;

**H3:** There is a positive relationship between knowledge management and entrepreneurial orientation.

#### Linking Intellectual Capital and Entrepreneurial Orientation

Prior literature indicates that intellectual capital positively influences entrepreneurial orientation (Monteiro et al., 2019; Poblete and Mandakovic, 2020). Entrepreneurial orientation, as a strategy, may facilitate the business through looking forward, garb the market opportunities, introducing the innovative products, differentiating the processes, and making a stable position of the organization (García-Villaverde et al., 2018). If intellectual capital capacities are utilized properly, they enhance human assets and organization abilities toward the entrepreneurial orientation attributes (Wach et al., 2018). Moreover, intellectual capital enhances the knowledgeable resources and capacities, that help to compete in an uncertain situation as well as improve the entrepreneurial orientation activities that help to take a right (Dabić et al., 2018; Martens et al., 2018). Human capital is linked with entrepreneurial orientation because high-quality human capital provides more knowledge that influences entrepreneurial orientation attributes. Such experience is considered essential to take an entrepreneurial decision proactively.

Social capital gives support to the development of entrepreneur activities; those organizations that have good social capital and entrepreneurial orientation behavior tend to have an influencing position in networks and thus these organizations have the tendency to grab more opportunities (Aljanabi, 2017). Prior studies indicate that intellectual capital has a positive impact on entrepreneurial orientation because as a knowledgeable resource of an organization, it enhances the entrepreneurial activities and is helpful in decision making (Guzmán et al., 2019).

The most relevant characteristic of the RBV is the focus on the internal forces of a firm, emphasis on the proper use of organizational resources, and creating and managing intangible organizational resources in a proper manner (Gupta et al., 2019). Intangible sources of organization and entrepreneurial orientation are essential to managing the several aspects and capabilities to compete in a dynamic market (Hock-Doepgen et al., 2021). According to RBV, the resources that are rare, valuable, difficult to imitate, and non-substitutability are the fundamental requirements of today’s firm to survive (Kollmann et al., 2016). Accumulating intellectual capital in a way to extend a the entrepreneurial orientation of a firm can be helpful for the survival of a firm (Alshanty and Emeagwali, 2019). Therefore, based on this discussion, the following hypothesis is established;

**H4:** There is a positive relationship between intellectual capital and entrepreneurial orientation.

#### Linking Entrepreneurial Orientation and Innovation

Entrepreneurial orientation refers to the strategic orientation of a firm and it represents how firms exploit knowledge-based resources to discover and exploit new opportunities (Rodrigo-Alarcón et al., 2018). Entrepreneurial orientation is the organizational capability to find out the unexplored opportunity and can accept the risk under dynamic circumstances, and innovation is also the organizational skill and capability to utilize the organization’s resources and transform that knowledge and resource for granting new products and processes (de Guimarães et al., 2018). Prior studies testify to the relationship between entrepreneurial orientation and innovation and found that innovation is the key indicator of entrepreneurial orientation (Arzubiaga et al., 2018; Zhai et al., 2018). As the above explained that the entrepreneurial orientation is the key element for entrepreneurial behavior that is conceived as a new venture and continually explored new opportunities by utilizing the existing knowledge and information. Entrepreneurs are more aware of the economic dynamics and take that dynamic as an opportunity, more aware of the new information and trends that lead to enhancing the innovation activity (Tang et al., 2012). Moreover, for innovation, an organization needs intensive and extensive knowledge that highly depends on an organizational workforce which analyzes the market trend on time and drives the organization toward innovation by utilizing organization resources (Genc et al., 2019).

Ferreira et al. (2020) argued that entrepreneurial orientation plays a crucial role in collecting resources and transferring them into enhancing innovation. According to the RBV perspective, the organization is a blend of various tangible and intangible resources and capabilities to manage these resources (Ciampi et al., 2021). The entrepreneurial orientation is one of the key capabilities to managing organizational resources because it is intended to gain a competitive advantage by utilizing resources and capabilities (Aljanabi, 2017). Therefore, entrepreneurial orientation analyzes the environmental dynamics and proactively takes the opportunity for innovation. Thus, based on this discussion, the following hypothesis is suggested;

**H5:** There is a positive relationship between entrepreneurial orientation and innovation.

#### The Mediating Role of Entrepreneurial Orientation

In this study, we propose a mediation mechanism of entrepreneurial orientation in the relationship between knowledge management, intellectual capital, and innovation. First, we discuss the mediating role of entrepreneurial orientation between the relationship of knowledge management and innovation. Several studies tried to test entrepreneurial orientation as a mediator like self-concept characteristics and performance, leadership style and performance (Al-Jinini et al., 2019), cultural background and performance (Soomro and Shah, 2019), and transformational and transactional leadership and performance (Arham et al., 2017). However, the existing literature has some mixed evidence showing the importance of entrepreneurial orientation in the relationship between knowledge management and innovation (Alvino et al., 2020; Ciampi et al., 2021).

Entrepreneurial businesses are more inclined to pursue a variety of opportunities and venture into a variety of fields (Isichei et al., 2020). Additionally, when businesses explore a variety of options, an entrepreneurial attitude can help them stand out (Montiel Campos, 2017). A high level of knowledge convergence has the ability to achieve strategic consistency and produce synergies between diverse businesses, resulting in improved organizational value. Without an effective framework for information integration, an entrepreneurial stance may not go very far. The following hypothesis is presented in light of the little research on entrepreneurial orientation as a mediator;

**H6 (a):** Entrepreneurial orientation mediates the relationship between knowledge management and innovation.

Second, the study discusses the mediating role of entrepreneurial orientation between the relationship of intellectual capital and innovation. Intellectual capital boosts the entrepreneur orientation activities because entrepreneur orientation decides on the base of organization resources, and intellectual capital is the intangible asset of the organization (Caseiro and Coelho, 2018). Moreover, Rezaei and Ortt (2018) explain that environmental factors influence entrepreneurial orientation proactively and take the opportunity to grab the highly dynamic environment. As a result, opportunities are raised; therefore, entrepreneurs take a risk and ultimately drive innovation (Genc et al., 2019). Teixeira et al. (2019) explain that innovation has the potential risk but it provides great benefits to the organization in return, but intellectual capital provides leverage for enhancing the entrepreneurial orientation activities which reduced the perceived risk in innovation and also increases the profitability of an organization.

Entrepreneur orientation develops organizational knowledge and the ability to make a decision. Kianto et al. (2017) argued that intellectual capital and entrepreneurial orientation are the internal capabilities of organizations that are also related to the external market condition and innovation. Inspiring from the entrepreneurship literature, one of the more significant findings to emerge, is that entrepreneurship comes to play as a lever to mobilize a company’s various knowledge resources more effectively and as the mediator between intellectual capital components and innovation (Kollmann et al., 2016; Poblete and Mandakovic, 2020). Moreover, the RBV addresses the issue of how to acquire and exploit distinct resources of an intellectual capital to achieve entrepreneurial orientation and innovation within a firm to enhance its competitive performance (Alshanty and Emeagwali, 2019). Thus, based on this discussion, entrepreneurial orientation plays a fundamental role in assembling resources and also converts them into enhancing innovation. Hence, the following hypothesis is established;

**H6 (b):** Entrepreneurial orientation mediates the relationship between intellectual capital and innovation.

#### Leader’s Education as Moderator

Existing studies tested different moderators like knowledge intensity as a moderator between knowledge processes and innovativeness (Andreeva and Kianto, 2011), innovation culture in the relationship between knowledge assets and product innovation (Denicolai et al., 2014), and convergence federation between knowledge management and innovation (Islam and Ikeda, 2014). Moreover, moderating effect of knowledge-centered culture, knowledge-oriented leadership, and knowledge-centered HR practices in the relationship between knowledge exploration and innovation outcomes of companies was tested by Lei et al. (2019), while social media capability between information technology (IT)-enabled knowledge ambidexterity and innovation performance was examined by Benitez et al. (2018). Similarly, the literature also suggests different moderators in the relationship between intellectual capital and innovation, such as dynamic capability between intellectual capital and innovative performance were investigated by Han and Li (2015), social capital and entrepreneurial orientation in the relationship between intellectual capital and innovation were tested by Wu et al. (2008), social capital between knowledge sharing and professionals’ innovative behavior were tested by Moustaghfir et al. (2013), social networks in the relationship between knowledge management and the radical innovation process, strategic knowledge management in the relationship between three components of intellectual capital, firm innovation, and market performance were examined by Cabrilo and Dahms (2018).

However, the above literature suggests that there are no or only a few studies that have investigated the impact of a leader’s education level on the proposed relationships. For example, Flanigan et al. (2017) and Li et al. (2019) investigated the demographic characteristics between leadership and small firm financial performance. However, there is some imprecise evidence of moderation of leaders’ education between the study variables. In the literature, the level of education for leaders in diverse organizations has garnered a lot of attention, with mixed outcomes. According to the study by Silva (2014), higher education is not required for the success of company leaders, but it can be beneficial to the success of leaders and organization. Higher levels of education for a CEO have been linked to increased corporate innovation in other research (Flanigan et al., 2017). Therefore, this study formulated the moderating effect of the leader’s education level in the relationship between intellectual capital and knowledge management and innovation:

**H7 (a):** A leader’s education will moderate the effect of knowledge management on entrepreneurial orientation such that higher levels of education will increase the strength of the positive association between knowledge management and entrepreneurial orientation.

**H7 (b):** A leader’s education will moderate the effect of intellectual capital on entrepreneurial orientation such that higher levels of education will increase the strength of the positive association between intellectual capital and entrepreneurial orientation.

**FIGURE 1:**
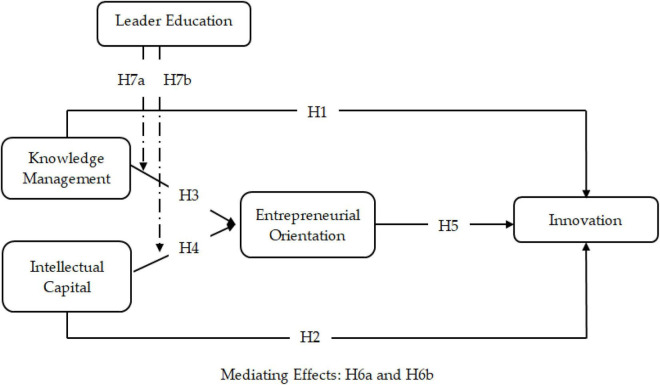
Conceptual model.

The conceptual model depicting the relationships is given in Figure 1.

## Materials and Methods

### Sample and Data Collection

This study used an exploratory research design. The nature of the study was cross-sectional and based on quantitative data. The study generated primary data through an email survey performed using a sample of Pakistani SMEs in the Information Technology (IT) sector. To select the sample, we chose IT firms registered in the company directory of the Pakistan Software House Association (PSHA). The PSHA has almost 896 active IT companies, which possess expertise in custom software development, enterprise resource planning (ERP), financial solutions, mobile content, document management, enterprise computing, and business process outsourcing. The major reason for selecting the IT firms is the high potential for innovation and greater contributions of these firms to economic development as well as in creating new opportunities for other business services. Moreover, intellectual capital and product innovation are more relevant for these companies than for service companies. The IT Sector of Pakistan is relatively well developed and has had a slightly better rate of growth in recent years. The data were collected through a structured email survey from the CEO, director of innovation, or from the top executives from July to August, 2020.

Following the procedure of Engelen et al. (2014) an email was sent to each company with an invitation to fill out a survey and a letter explaining our research background and purpose. We have taken a number of steps to boost response rates, including allowing the participants to complete the survey offline and return it via regular mail, fax, or email; providing respondents with a report of our survey results, which includes descriptive statistics and anonymous comparisons of participating companies; and sending two personalized reminder emails to all potential respondents. Finally, we received results from 410 of 896 companies that are invited to complete the survey (45.75% response rate). We eliminated 17 surveys that were incomplete and as a result, 393 responses remained for data analysis. Firm age is measured using the experience of the firm. For the classification in terms of the size of the SME, we followed the study by Bojica and Fuentes (2012). Bojica and Fuentes referred to the EU’s Directive 78/660/CEE, which considers small firms to be those with fewer than 50 employees and medium-sized firms to be those with fewer than 250 employees.

Table 1 present the demographic characteristics of the sample. A total of 393 firms participated in this study, 8.9% of firms’ age is 1–3 years, 22.1% of firms’ age is between 4 and 7 years, 33.8% of participants firms’ age is 8–10 years, and 28.2% firms’ age is above 10 years. Moreover, 49.6% of the questionnaires were answered by the CEOs, and 42.0% were responded to by directors/manager innovation. Furthermore, 8.4% were answered by top managers, who report directly to the CEO. Respondent’s education data show that 42.5% of respondents have bachelor’s degrees and 9.9% have a Ph.D. and 10.9% have other qualifications.

**TABLE 1:** Sample characteristics.

Age of firm	Frequency	Percent
1–3 years	43	10.9
4–7 years	106	27.0
8–10 years	133	33.8
Above 10 years	111	28.2
**Firm size**	**Frequency**	**Percent**
10–50 employees	175	44.5
51–100 employees	129	32.8
101–250 employees	64	16.3
Above 250 employees	25	6.4
**Respondents positions**	**Frequency**	**Percent**
CEO	195	49.6
Director/Manager innovation	165	42.0
Other top Managers	33	8.4
**Respondents education**	**Frequency**	**Percent**
Bachelors	167	42.5
Masters	144	36.6
MS/Ph.D	39	9.9
Other	43	10.9
**Total**	**393**	**100.0%**

### Measures

#### Knowledge Management Process

The knowledge management process is a combination of three elements including knowledge acquisition, knowledge sharing, and knowledge utilization practices (Appendix). This study adopted nine items measurement scale of knowledge management from the study of Obeidat et al. (2016) that aggregates three dimensions of knowledge management, such as acquisition, sharing, and utilization. A sample item for knowledge acquisition is, “we hire new employees as a source for acquiring new knowledge.” A sample item for knowledge sharing is, “we share information and knowledge necessary for the tasks.” A sample item for knowledge utilization is, “the firm utilizes available knowledge to improve its performance.” All items were measured on a 5-point Likert scale, ranging from 1 for strongly disagree to 5 for strongly agree.

#### Intellectual Capital

The intellectual capital was measured using a 5-point Likert scale with the help of two dimensions, such as human and social capital. Human capital was evaluated through five items scale adapted from the study of Al-Jinini et al. (2019). A sample item is, “our employees are highly skilled.” Moreover, to measure the social capital, we adapted four items scale from the study of Al-Jinini et al. (2019). A sample item is, “our company documents its projects to use it in other projects.”

#### Entrepreneurial Orientation

This study operationalized entrepreneurial orientation through innovativeness and proactiveness. Entrepreneurial orientation was rated using a 5-point Likert scale. To measure innovativeness and proactiveness, we adopted five measurement constructs from the study by Al-Jinini et al. (2019). A sample item for innovativeness is, “employees are motivated to think and perform innovatively.” A sample item for proactiveness is, “our company offer a new products more than their competitors.”

#### Innovation

This study conceptualizes innovation as two-dimensional constructs, such as product innovation and process innovation. Innovation was evaluated using 5-point Likert scales. Moreover, to measure product and process innovation, we adopted 8-items scale from the study by Al-Jinini et al. (2019). A sample item for product innovation is, “our company introduces modifications to its existing product and services.” A sample item for process innovation is, “the company work processes are constantly updated.”

#### Leaders’ Education

To measure leaders’ education, we used 5-points Likert scale and 6 items were adapted from the study by Más-Machuca (2014). A sample item is, “our company leaders educate employees to encourage knowledge sharing in the organization.”

## Results

### Data Analysis and Results

The data were analyzed using the Smart-PLS software 3.0 version. This software is currently considered one of the suitable software to apply partial least squares structural equation modeling (SEM) (Sarstedt and Cheah, 2019). PLS-SEM was recommended in most business management studies (Ringle and Sarstedt, 2016). The method is preferred for theory testing and confirmation and is appropriate for checking the existence of complex relationships (Hair et al., 2017). The PLS-SEM allows for the construction of a research paradigm based on a theory that involves transforming theories and concepts into unmeasured variables (latent) and practical concepts into metrics, all of which are connected by a theory or hypothesis (Becker et al., 2012). Cheah et al. (2018) suggest that the PLS-SEM model should be assessed in three phases: identifying the global model assessment, checking the measurement model’s validity, and analyzing the relevance of the routes inside the SEM.

### Measurement Model

To test the reliability of constructs, Cronbach’s alpha, composite reliability, and AVE values were assessed. According to Henseler and Fassott (2010), the criterion for ensuring the composite reliability (CR) is that all values must be higher than 0.7. All values of composite reliability are presented in Table 2. The values lie between the ranges of 0.944 and 0.963, which confirms the composite reliability of all of the constructs. Moreover, the Cronbach’s alpha for all constructs was also above the threshold value of 0.7 suggested by Hair et al. (2011). Furthermore, the average variance extracted (AVE) criterion allows its value to be greater than 0.50 (Fornell and Larcker, 1981). Therefore, Table 2 shows that the AVE values of the constructs ranged from 0.714 to 0.811 and met the criteria. Prior researchers argue that if the values of AVE are above the acceptable level of 0.5, it indicates adequate convergent validity (Bagozzi and Yi, 1988; Henseler and Fassott, 2010).

**TABLE 2:** Measurement model.

Variable and constructs	Loadings	α	CR	AVE	VIF
**Entrepreneurial orientation**		**0.942**	**0.955**	**0.811**	
INO1	0.895				3.390
INO2	0.905				3.804
INO3	0.847				2.819
PRO1	0.929				4.627
PRO2	0.924				4.357
**Innovation**		**0.956**	**0.963**	**0.765**	
INN1	0.814				2.963
INN2	0.901				3.195
INN3	0.867				3.407
INN4	0.874				3.441
INN5	0.893				4.878
INN6	0.863				3.182
INN7	0.861				3.484
INN8	0.920				2.381
**Intellectual capital**		**0.950**	**0.957**	**0.714**	
HC1	0.864				3.380
HC2	0.819				2.769
HC3	0.856				3.276
HC4	0.809				2.656
HC5	0.817				2.734
SC1	0.857				3.293
SC2	0.874				3.877
SC3	0.890				4.155
SC4	0.817				2.741
**Knowledge management**		**0.953**	**0.960**	**0.725**	
KA1	0.925				5.504
KA2	0.858				3.805
KA3	0.877				3.858
KS1	0.850				2.801
KS2	0.853				3.823
KS3	0.867				3.873
KU1	0.769				2.328
KU2	0.853				3.389
KU3	0.805				2.597
**Leader education**		**0.931**	**0.944**	**0.741**	
LE1	0.907				3.405
LE2	0.648				1.642
LE3	0.845				3.066
LE4	0.939				5.788
LE5	0.925				5.198
LE6	0.868				2.798

*α, Cronbach’s alpha; CR, Composite Reliability; AVE, Average Variance Extracted; VIF, Variance Inflation Factor.*

Additionally, discriminant validity was calculated using the Fornell–Larcker criterion as findings are expressed in Table 3. The findings show that most of the correlations of the constructs with each other are fewer than the square roots of their AVEs, demonstrating that our measures have discriminant validity (Fornell and Larcker, 1981). Besides, discriminant validity was also assessed using Heterotrait-Monotrait ratio (HTMT) criteria (Henseler et al., 2014). The results listed in Table 4 indicate that HTMT values were satisfactory and below the threshold of 0.85 as suggested by Hair et al. (2011). We also calculated the variance inflation factors (VIFs) for all constructs in our model to test for multicollinearity. All VIF values were below 5.788, lower than the threshold of 10, indicating no concerns regarding multicollinearity issues in the data (Ringle and Sarstedt, 2016). Finally, Harman’s single factor test was used to check for common method bias in the data. According to Harman’s technique, common method bias exists when one component emerges from factor analysis and explains more than 50% of the variance (Podsakoff, 2003). We used the rotated solution to transfer all the items into a one-factor analysis, yielding four factors; the first factor’s eigenvalue explains 29.38 percent of the variance (50 percent). As a result, it is clear that this study does not suffer from common technique bias.

**TABLE 3:** Fornell-Larcker criterion.

	Entrepreneurial orientation	Innovation	Intellectual capital	Knowledge management	Leader education
Entrepreneurial orientation	0.901				
Innovation	0.424	0.875			
Intellectual capital	0.299	0.317	0.845		
Knowledge management	0.221	0.291	0.138	0.852	
Leader education	0.141	0.027	0.083	-0.002	0.861

*Items with diagonals are the square root of the AVE. Items under diagonals are the correlations.*

**TABLE 4:** Heterotrait-Monotrait Ratio (HTMT) criterion.

	Entrepreneurial orientation	Innovation	Intellectual capital	Knowledge management	Leader education
Entrepreneurial orientation					
Innovation	0.439				
Intellectual capital	0.308	0.323			
Knowledge management	0.229	0.288	0.141		
Leader education	0.133	0.075	0.094	0.046	

### Structural Model

**FIGURE 2:**
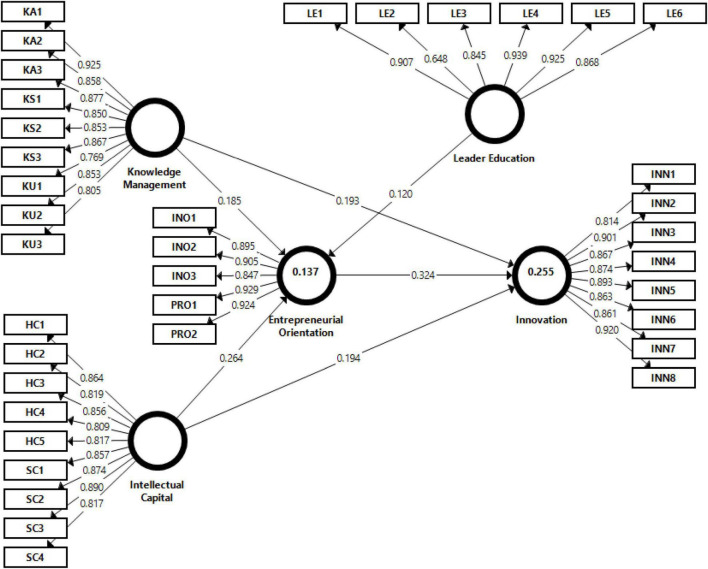
Measurement model.

The structural model was evaluated through the 5,000 bootstrap method with the help of Smart-PLS software. The fitness of the structural model was assessed by the standardized root mean squares residual SRMR value. According to Henseler and Fassott (2010), a good structural model should have a value below the 0.08 SRMR value. Therefore, the findings from the structural model show a 0.049 value of SRMR that was acceptable below the threshold. Moreover, to assess the value of *R*^2^, the structural model explained (13.7%) variance in entrepreneurial orientation and (25.5%) variance explained in innovation. As suggested by prior researchers the value of *R*^2^ and *Q*^2^ should be greater than 0.10 or zero (Hair et al., 2011; Henseler et al., 2014). Furthermore, the results provided in Table 5 and Figure 2 indicate that the values of *R*^2^ and *Q*^2^ are greater than the threshold value. Hence, the structural model was acceptable and met the criteria for further analysis.

**TABLE 5:** Path coefficients (direct effects).

Hypotheses	Relationships	β	*t*	*p*	*R*^2^ = Entrepreneurial orientation 0.137; Innovation = 0.255 *Q*^2^ = Entrepreneurial orientation 0.106; Innovation = 0.188
H1	Knowledge management → Innovation	0.193	3.649	0.000	
H2	Intellectual capital → Innovation	0.194	3.433	0.001	
H3	Knowledge management → Entrepreneurial orientation	0.189	3.223	0.001	
H4	Intellectual capital → Entrepreneurial orientation	0.233	4.326	0.000	
H5	Entrepreneurial orientation → Innovation	0.324	5.755	0.000	
	Leader education → Entrepreneurial orientation	0.110	2.483	0.013	

### Hypothesis Testing

**FIGURE 3:**
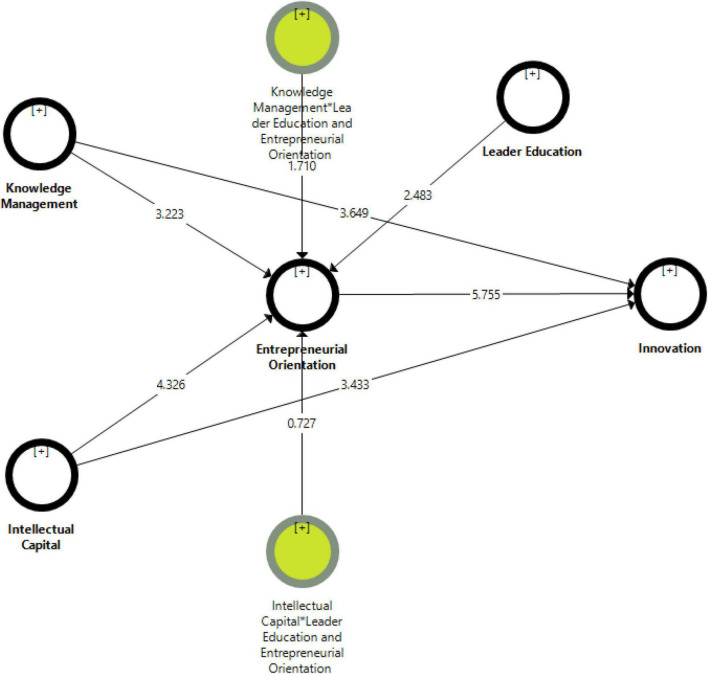
Bootstrapping.

The study analyzed the hypotheses with bootstrapping to explore the significance level between all the variables. All the hypotheses were statistically significant and the results are shown in Table 6 and Figure 3. The findings of H1 indicate that knowledge management has a positive and significant effect on innovation with a standardized path coefficient (0.193^**^, *t* = 3.649, *p* = 0.001). Therefore, H1 was accepted. Moreover, the results of H2 show that intellectual capital has a positive and significant influence on innovation with a standardized path coefficient (0.194^**^, *t* = 3.433, *p* = 0.001). Thus, H2 was supported. Furthermore, the findings of H3 illustrate that knowledge management has a positive and significant impact on entrepreneurial orientation with a standardized path coefficient (0.189^**^, *t* = 3.223, *p* = 0.001). Hence, H3 was accepted. Additionally, the results of H4 show that intellectual capital has a positive and significant effect on entrepreneurial orientation with a standardized path coefficient (0.223^**^, *t* = 4.326, *p* = 0.001). Therefore, H4 was supported. Meanwhile, we tested H5 and the findings explore the entrepreneurial orientation that has a positive and significant impact on innovation with a standardized path coefficient (0.324^**^, *t* = 5.755, *p* = 0.001). Hence, H5 was accepted.

**TABLE 6:** Specific indirect effect.

Hypotheses	Relationship	β	*t*	*p*
H6a	Knowledge management → Entrepreneurial orientation → Innovation	0.061	2.926	0.003
H6b	Intellectual capital → Entrepreneurial orientation → Innovation	0.075	3.565	0.000

Besides, we tested the indirect effect of entrepreneurial orientation in the relationship between knowledge management and intellectual capital on innovation. The results of H6a and H6b were presented to indicate that entrepreneurial orientation has an indirect positive and significant influence on the related knowledge management and intellectual capital on innovation with a standardized path coefficient (0.061^**^, *t* = 2.926, *p* = 0.001; 0.075^**^, *t* = 3.565, *p* = 0.001). Likewise, to assess partial and full mediation effects, we followed the approach by Hair et al. (2017) approach using the variance accounted for VAF and analyzed the direct, indirect, and total effects. According to this method, if the value of VAF is between 20 and 80%, it presents partial mediation and if the value of VAF is more than 80%, a full mediation exists between the variables. Thus, the findings listed in Table 7 show that the value of VAF is below 80%, which presents partial mediation. Hence, H6a and H6b were accepted.

**TABLE 7:** Mediation analysis (entrepreneurial orientation as a mediator).

Exogenous variable	Direct effect	Indirect effect	Total effect	VAF (%)	Mediation	Endogenous variable
Knowledge management	0.189	0.061	0.250	24.4%	Partial mediation	Entrepreneurial orientation
Intellectual capital	0.233	0.075	0.308	24.3%	Partial mediation	Entrepreneurial orientation

Lastly, we assessed the moderating effect of a leader’s education in the relationship between knowledge management and intellectual capital on entrepreneurial orientation. The findings are expressed in Table 8 and show that leader education strengthens the association between knowledge management and entrepreneurial orientation with a standardized path coefficient (0.097^**^, *t* = 1.710, *p* = 0.042). Thus, H7a was supported. Furthermore, H7a findings mention that leader education strengthens the association between intellectual capital and entrepreneurial orientation with a standardized path coefficient (−0.040^**^, *t* = 0.727, *p* = 0.467). Thus, H7b was not accepted.

**TABLE 8:** Moderating effect (leader education as a moderator).

Hypotheses	Relationship	β	*t*	*p*	Decision
H7a	Knowledge management × Leader education and entrepreneurial orientation	0.097	1.710	0.042	Yes
H7a	Intellectual capital × Leader education and entrepreneurial orientation	−0.040	0.727	0.467	No

## Discussion and Conclusion

### Discussion

The knowledge-based view shows that as a rare, unique, valuable, and irreplaceable resource (Iqbal et al., 2018), knowledge is the most critical asset for enterprises to establish, share, and systematize sustainable competitive advantage. Researchers have believed that intellectual capital is a key factor to boost the innovation and performance of the firm (Subramaniam and Youndt, 2005), especially for those SMEs which may have difficulties in competing in the market (McDowell et al., 2018). In addition, as knowledge management requires knowledge acquisition, communication, and compilation (Teixeira et al., 2019), researchers recommend that companies use knowledge through strategic capabilities, such as innovation to improve corporate performance (Bojica and Fuentes, 2012; Roxas et al., 2013). Based on this view, we studied the mediating effect of entrepreneurial orientation on the relationship between knowledge management, intellectual capital, and innovation.

The first objective of the current study is to find the statistical relationship between knowledge management, intellectual capital, and innovation in IT sector firms of Pakistan. Prior literature suggests that knowledge management and intellectual capital are significantly important for SMEs and IT sector firms, in particular. But knowledge management and intellectual capital are not well researched in the IT sectors of Pakistan. Therefore, based on prior literature and understandings, the researchers have developed and proposed the existence of a statistical relationship between knowledge management, intellectual capital, and innovation. This proposition is tested and verified through the empirical data and found significant, as is supported in previous studies (López-Nicolás and Meroño-Cerdán, 2011; Sarwat and Abbas, 2020) on knowledge management and innovation (Han and Li, 2015; Buenechea-Elberdin et al., 2017; Li et al., 2019) as well as on intellectual capital and innovation.

This study aims to test the relationship between knowledge management, intellectual capital, and entrepreneurial orientation in the IT sector of Pakistan. The role of intellectual capital practices in terms of human and social capital are the important factors within the organization to trigger the entrepreneurial orientation. This research recognizes that appropriate knowledge management processes and intellectual capital practices among employees provide the firm with ultimate support in the industry. These study findings revealed a positive and significant relationship between knowledge management and entrepreneurial orientation similar to the study by Arzubiaga et al. (2018) and Al-Jinini et al. (2019), and between intellectual capital and entrepreneurial orientation. A similar result is also reported in previous studies (Wu et al., 2008; Dabić et al., 2018).

Measuring the postulated relationship between entrepreneurial orientation and innovation is also the aim of the study. The study found a significant and positive relationship between entrepreneurial orientation and innovation. Previous literature suggests that an improvement in the entrepreneurial orientation of a firm can promote innovation. These findings are in line with the prior studies (Masa’deh et al., 2018; Alrowwad et al., 2020). For the survival and embrace of the innovation in the organization, there is a need to move according to the changing market by adopting new technology, learning new skills, and also making a blend of all resources, such as internal or external that improve the innovation (Amin and Aslam, 2017).

This research requires measuring the anticipated effects of entrepreneurial orientation as a mediator among knowledge management, intellectual capital, and innovation. The results suggest that entrepreneurial orientation proves as an area of substantial importance in promoting innovation in the IT sector of Pakistan. These findings argue that entrepreneurial orientation partially mediates the relationship between knowledge management, intellectual capital, and innovation, and the results are in line with the past literature (Rezaei and Ortt, 2018; Mostafiz et al., 2021).

The current study considers the moderating role of a leader’s education in the relationship between knowledge management, intellectual capital, and entrepreneurial orientation in IT sector firms in Pakistan. The results show that a leader’s education significantly moderates the effect of knowledge management on entrepreneurial orientation. However, a leader’s education does not moderate the effect of intellectual capital on entrepreneurial orientation such that low levels of education will decrease the strength of the positive association between intellectual capital on entrepreneurial orientation. This finding is in line with the prior study by Flanigan et al. (2017).

### Conclusion

This manuscript contributes to the entrepreneurial orientation and literature on knowledge management, intellectual capital, and innovation. The research outlines the theoretical perspective that knowledge management and intellectual capital, support a firm to extend and deploy its entrepreneurial orientation to improve its innovation, especially if the firm operates in a turbulent market. In so doing, the study shows that the firm should use some consistent strategies for knowledge management and intellectual capital accumulation to build the desired innovation capacities. The theoretically derived research model, which links knowledge management, intellectual capital, and innovation, and also the moderating role of a leader’s education was empirically validated using an empirical study of 393 small and medium-sized firms in the IT sector of Pakistan.

## Implications

### Theoretical Contributions

The study has advanced the theoretical contribution regarding knowledge management, intellectual capital, innovation, and entrepreneurship research in the following aspects. First, the research introduced knowledge management as an antecedent of entrepreneurial orientation and innovation to cover the existing gap. Moreover, this study measures intellectual capital as a first-order construct distinguishing it in human capital and social capital. Second, this study examined entrepreneurial orientation as a mediator between knowledge management, intellectual capital, and innovation, which was previously, not discussed by the prior researchers. Third, this research introduces a leader’s education as a moderator between knowledge management-entrepreneurial orientation and intellectual capital, entrepreneurial orientation relationships that were previously ignored in the relevant literature.

Another notable contribution of this study is that it examined a mediator that was otherwise considered an independent variable traditionally and answered whether entrepreneurial orientation positively mediates the relationships between knowledge management process and intellectual capital on innovation. In this sense, the findings of the study demonstrated that entrepreneurial orientation characteristics, such as risk-taking, innovativeness, and proactiveness, are critical to the effective deployment of knowledge application and innovation. Furthermore, this study contributes to the existing literature as it examines the relationship in a context of a collectivistic country as these conceptions are earlier developed and tested in Latin America and Western culture (Rosenbusch et al., 2011; McDowell et al., 2018). Hence, this study was conducted in the IT sector of Pakistan to fill the gap as suggested by Agostini et al. (2017) that future researchers could add more contributions to investigate the intellectual capital and innovation relationship in different sectors to increase the generalizability of constructs.

### Practical Implications

Moreover, this study provides implications for managers, entrepreneurs, and policymakers who are directly involved particularly in the IT sector of Pakistan. Knowledge management and intellectual capital seem to be more beneficial for firms with high levels of intellectual capital accumulation and knowledge-based resources. However, managers have to craft appropriate resource combinations to take advantage of both intellectual capital and knowledge to leverage the innovation and to translate it into superior firm performance. The creation of intellectual capital and knowledge help firms in harnessing product and process innovation as well as facilitates the firms to make it sustainable. To promote intellectual capital and knowledge management to get competitive advantages, managers can use different entrepreneurial mechanisms. Moreover, by focusing on intellectual capital, knowledge management, and entrepreneurial orientation, firms can set the grounds for generating new, innovative, and creative ideas as well as thoughts to be innovative. As the study recommends the mediating impact of entrepreneurial orientation, the owners and managers in SMEs, particularly IT firms are required to commit their complete potential to ensure entrepreneurial orientation and also motivate their employees to practice it.

Furthermore, the study provides insights into the role of a leader’s education in promoting entrepreneurial orientation and innovation, particularly for SMEs who practice intellectual capital might improve innovation in their companies if they have a more qualified leader. As the innovation index shows that Pakistan is occupying a low position, the firms should hire more educated leaders to implement a successful innovation process in SMEs. The findings of the research are also important from a social perspective, particularly for the accumulation of social capital. Concerning intellectual capital development, the firms that form social capital are more likely to get success. Evidence for the argument can be found in the knowledge-intensive SMEs that are evident of heavy investment in resources, such as physical assets, strong relationships at personal as well as a team level, maintaining high trust levels, control norms, and strong networks across permeable limits. An investment in social capital enables a firm to foresee the market changes and meeting with customer demands more appropriately. Thus, understanding the social perspective helps managers to understand the social ties and invest in corporate social responsibility at the appropriate time.

### Limitations and Future Research Directions

This study provides some limitations and future research directions for upcoming researchers. First, the study is cross-sectional; so the underlying analyses of results could be vulnerable. However, a longitudinal analysis is required to examine and elucidate the postulated relationship among study variables at different times. Second, the limitation addresses how the variables are measured. The study uses self-reported measures, raising probable issues of common method biases. However, self-reported questions are found to be more appropriate to measure the firm responses to the study variables. To address the common method biases issue, VIF is calculated using the procedure prescribed by Podsakoff (2003). This theoretical model is generalized in the information technology industry and therefore another scholar may generalize this model in Pakistan in any other sector and it has the potential to be generalized in any other under developing country for boosting the business performance. The single industry study is one more limitation of this study. Future studies can add emotional intelligence or leader’s efficacy as a moderator. Moreover, the future study may testify to this model with actor-network theory.

## Data Availability Statement

The raw data supporting the conclusions of this article will be made available by the authors, without undue reservation.

## Ethics Statement

The studies involving human participants were reviewed and approved by National Textile University, Faisalabad, Pakistan. The patients/participants provided their written informed consent to participate in this study.

## Author Contributions

QY and SA proposed the research, analyzed the results, and wrote the manuscript. WJ, MM, and NS revised the whole manuscript and extensively edited the manuscript. All authors contributed to the article and approved the submitted version.

## Conflict of Interest

The authors declare that the research was conducted in the absence of any commercial or financial relationships that could be construed as a potential conflict of interest.

## Publisher’s Note

All claims expressed in this article are solely those of the authors and do not necessarily represent those of their affiliated organizations, or those of the publisher, the editors and the reviewers. Any product that may be evaluated in this article, or claim that may be made by its manufacturer, is not guaranteed or endorsed by the publisher.

## Appendix

### Knowledge Management Process

#### Knowledge Acquisition

KA1: We hire new employees as a source for acquiring new knowledge.

 KA2: We provide an open environment to help our employees acquire new knowledge.

 KA3: We continually gather information that is relevant to our operations and activities.

##### Knowledge Sharing

KS1: We share information and knowledge necessary for the tasks.

 KS2: We exchange knowledge between employees in order to achieve our goals with little time and effort.

 KS3: We promote sharing of information and knowledge between team members and various units.

##### Knowledge Utilization

KU1: The firm effectively manages various sources and types of knowledge.

 KU2: The firm utilizes available knowledge in improving services provided to its customers.

 KU3: The firm applies available knowledge in order to improve its performance.

#### Intellectual Capital

##### Human Capital

HC1: Our employees are highly skilled.

 HC2: Our employees are widely considered the best in our industry.

 HC3: Our employees are capable of developing new ideas in their jobs.

 HC4: Our employees are considered experts in their particular jobs and functions,

 HC5: Our employees develop new ideas and knowledge.

##### Social Capital

SC1: Our company uses intellectual property rights (patents/registered software, and copyrights) as a way to store knowledge.

 SC2: Our company protects knowledge and key information to avoid loss if key people left the company.

 SC3: Our company documents its projects in order to use it in other projects.

 SC4: Our company possesses work methods and procedures in support of innovations and new products.

#### Entrepreneurial Orientation

##### Innovativeness

INO1: Our company empowers its employees to try on new ideas regardless of their job position.

 INO2: Employees are motivated to think and perform innovatively.

 INO3: Imitating the innovation of another corporation is a company’s innovation.

##### Proactiveness

PRO1: Our company offers new products more than their competitors.

 PRO2: Our company emphasizes the importance of creating new innovative products.

#### Innovation

INN1: Our company introduces modifications to its existing product or services.

 INN2: Our company constantly develops new products or services.

 INN3: The companies’ new products and services are often perceived as novel by customers.

 INN4: In new products and service introductions, our company is often first-to-market.

 INN5: In comparison with the company’s competitors, our company has introduced more innovative products and services during the past years.

#### Process Innovation

INN6: The work processes of our company are constantly updated.

 INN7: Our company emphasizes the development of new ways to provide its services.

 INN8: Our company constantly uses up-to-date technology to enhance products and services.

#### Leaders’ Education

LE1: Our company leaders educate employees to encourage knowledge sharing in the organization.

 LE2: Our company leaders support employees in getting an education.

 LE3: Our company leaders educate the employee in an effective way.

 LE4: Our company leaders educate the employees to generate innovative ideas.

 LE5: Our company leadership has the ability to manage a work a time.

 LE6: Our company leadership is highly qualified.
